# A safety study of laparoscopic single-anastomosis duodeno-ileal bypass with gastric plication (SADI-GP) in the management of morbid obesity

**DOI:** 10.1007/s00423-021-02276-9

**Published:** 2021-08-17

**Authors:** Istvan Bence Balint, Ferenc Csaszar, Lajos Orban, Peter Radics, Akos Farics, Gergo Manfai, Veronika Hari, Rebeka Javor

**Affiliations:** 1 Department of General and Vascular Surgery, Zala County Saint Rafael Hospital, Zrínyi Miklós street, 8900 Zalaegerszeg, Hungary; 2 Doctoral School of Neurosciences, University of Pecs, Pécs, Hungary; 3 Department of Psychiatric Rehabilitation, Szigetvár Hospital, Szigetvár, Hungary; 4 Department of General Surgery, Kanizsai Dorottya Hospital, Nagykanizsa, Hungary; 5 Central Anaesthesiological and Intensive Care Unit, Kanizsai Dorottya Hospital, Nagykanizsa, Hungary; 6 Department of Nutrition, Kanizsai Dorottya Hospital, Nagykanizsa, Hungary; 7 Faculty of Humanities and Social Sciences, Department of Community and Social Studies, University of Pécs, Pécs, Hungary

**Keywords:** Bariatric surgery, Metabolic surgery, Single-anastomosis duodeno-ileal bypass, One-anastomosis duodeno-ileal bypass, Omega-loop duodeno-ileal bypass, Gastric plication, Greater curvature plication, Minnesota Multiphasic Personality Inventory 2, Psychological evaluation, Obesity

## Abstract

**Background:**

Bariatric surgery is more effective in the management of morbid obesity and related comorbidities than is conservative therapy. Pylorus-preserving single-anastomosis duodeno-ileal bypass with sleeve gastrectomy (SADI-SG) is a modified duodenal switch technique. Gastric plication (GP) is an alternate to SG.

**Methods:**

Morbidly obese (BMI of > 40, or > 35 in the presence of diabetes or prediabetes) patients were recruited and operated on to perform SADI with GP. Complications related to surgery were recorded to assess the feasibility of the procedure. Weight-loss outcomes were analysed to determine efficacy. Minnesota Multiphasic Personality Inventory 2 (MMPI-2) was recorded after 1 year of follow-up, and test scales were used to describe physiological phenomena.

**Results:**

Seventeen middle-aged (mean: 40 years) patients were involved in our study; 15 of them were females. The mean duration of surgery was 205 min. There were no complications of conversion, death, bleeding, VTE or 30-day readmission to hospital. We did experience CD4a (pulmonary insufficiency due to chronic lung disease) and a CD3b (anastomosis leakage treated laparoscopically) complications. Vomiting occurred in three cases (CD1). Obesity-related comorbidities showed favourable resolution rates (77.8% for hypertension, 81.2% for dyslipidaemia, 100% for diabetes at the 1-year follow-up). Weight-loss outcomes were favourable (53.20 EWL%, and 35.58 TWL% at 1-year follow-up). Greater weight loss caused significantly higher levels of Depression (*t*(13.958) =  − 2.373; *p* = 0.00; *p* < 0.05) and Low Positive Emotions (*t*(13.301) =  − 2.954; *p* = 0.00; *p* < 0.05) and Introversion/Low Positive Emotionality (*t*(13.408) =  − 1.914; *p* = 0.02; *p* < 0.05) in MMPI-2 data.

**Conclusion:**

According to our safety study, SADI-GP is a promising malabsorptive procedure, but a long-term high-volume case series or a randomised controlled trial is necessary to evaluate complication rates and weight-loss outcomes. Emotional dysregulation is common among bariatric surgery patients according to personality inventory data; therefore, psychological follow-up and psychotherapeutic support are necessary for weight-loss maintenance.

**Supplementary Information:**

The online version contains supplementary material available at 10.1007/s00423-021-02276-9.

## Introduction

### Background

Morbid obesity is in correlation with hypertension, dyslipidaemia, prediabetes and T2DM [1]. If lifestyle modifications failed, bariatric surgery would be advised to morbidly obese patients [2–8]. LAGB, LSG and LGP are restrictive procedures [2–24]. BPD, SADJ-SG or SADI-SG, LRYGB and OAGB are malabsorptive surgical techniques [25–42]. LGP combined with omega-loop duodenal switch was described by Karcz et al. [43]. An experimental model described the superiority of SADJ-GP over SG in terms of efficacy in the remission of T2DM [44]. Two prospective observational studies reporting outcomes of a combination of LGP and OAGB were identified during a literature search [45, 46].

SADJ-SG and SADI-SG seem to have advantages over gastric bypass procedures (LRYGB and OAGB) because a more physiologic anastomosis (duodeno-jejunal or -ileal) is performed. Therefore, anastomotic ulcers deriving from afferent limb bile reflux through gastro-jejunal anastomosis should occur less frequently [27–35]. SG is the first part of these procedures which is very popular among bariatric surgeons due to its simplicity; however, it is only a restrictive method without the advantages of exclusion duodeno-jejunal mucosa from digestion. The major concerns of LSG are bleeding, leakage and augmented symptoms of GERD after surgery [2–7, 15, 19, 20, 22, 24]. During LGP, greater curvature of the stomach is invaginated into the gastric lumen in two or three folds by two layers of laparoscopic interrupted or running sutures. Gastric release, dilatation and prolapse (probably synonyms of the same effect in different stages) are common complications after LGP instead of bleeding and leakage. Gastric necrosis or perforation occurs rarely. Nausea and vomiting are associated with LGP very frequently [10–24].

### Objective

This paper presents the short-term results of the LASAGNE trial (LAparoscopic Single-Anastomosis duodeno-ileal bypass with Gastric plication (SADI-GP) in the maNagEment of morbid obesity) registered under number ISRCTN12800723 and approved by the institutional review board of Kanizsai Dorottya Hospital (hospital of Nagykanizsa) under protocol number KDK-2071–2/2019. The study is aimed to assess the safety and efficacy of SADI-GP based on a prospective cohort of patients recruited consecutively. We believe that combining well researched SADI with GP instead of SG could result in better complication rates and plausible metabolic outcomes.

## Methods

### Study design and setting

This trial used a cohort of consecutively admitted patients aiming at the assessment of complication rates and efficacy of SADI-GP. Patient recruitment began in October 2018 and ended in June 2019 (the last operation was performed in November 2019). Preoperative evaluation followed by surgery and postoperative follow-up visits (at 1, 3, 6 and 12 months) were planned.

### Participants

Patients between 18 and 65 years, with BMIs of > 40 (without comorbidity related to morbid obesity) or > 35 (with comorbidity related to morbid obesity, especially glucose metabolism) were included. Patients who had previous bariatric surgery, who had severe mental disorders (drug addiction, alcohol consumption, the use of antipsychotics), who were regarded as socially vulnerable patients, who were completely immobile, who were unable to understand the purpose of the study and bariatric surgery, who denied or withdrew their informed consent, for whom duodeno-ileal bypass was not performed during surgery or who dropped out before the 6-month follow-up were excluded.

### Variables

Primary endpoints of the study were to assess the safety of the investigated method and evaluate the risks due to surgery. Secondarily, weight-loss outcomes were analysed.

### Data source

The previous history, including the presence of hypertension, prediabetes, T2DM, dyslipidaemia, gout, cardiovascular disease, pulmonary disease, chronic venous disorder, PCO, OSA, GERD, peptic ulcer, osteoarthritis and mental disorders, was obtained. Body weight, height, BMI adjusted for age-gender-ethnicity, ideal weight (BMI of 25), excess weight and excess BMI were measured. Preoperative examinations (transthoracal cardiac ultrasound, abdominal ultrasound, carotid duplex ultrasound, spirometry, oesophago-gastro-duodenoscopy) were carried out. Blood tests (blood count, ionogram, serum protein, glucose, HgbA1C%, iron-binding capacity, lipid profile, kidney and liver function and haemostasis) were planned at baseline and at each follow-up visit. QoL was measured by BAROS-Moorehead-Ardelt II and Weiner et al. questionnaires [47, 48]. Postoperative complications were classified by Clavien-Dindo [49, 50].

### Procedure

Patients were operated in French position. The operating surgeon was positioned between the patient’s legs and one assistant surgeon was placed on each side. Under combined (general and peridural) anaesthesia, a skin incision was made one span (15–20 cm) under the xiphoid process, then the abdominal wall was prepared and lifted directly to prepare carbon dioxide peritoneum with a Veres needle, and an optic trocar (15 mm) was placed into the intra-abdominal cavity. Under visualisation, ports were created left (5–10 mm) and right (5–12 mm) of the umbilicus in the mid-clavicular line under the xiphoid process (10–12 mm depending on the type of liver retractor) and under the left rib arc in the front axillary line (5 mm). A liver retractor was placed through the epigastrial port, and the left upper abdomen was explored. The gastrocolic and gastrolienal ligaments were dissected besides the stomach by electrosonic cutting coagulation device (Thunderbeat, Olympus Co., Japan). A 54-F bougie was placed into the gastric lumen and guided through the pylorus. Depending on the anatomical situation, a two-layer threefold 2/0 polydioxanone (Polydox, Chirana, Czech Republic) running suture was made from fundus to antrum or a 2/0 polydioxanone (Polydox, Chirana, Czech Republic) running suture was made from fundus (one-layer onefold) through the corpus (two-layer threefold) to the antrum (one-layer onefold). The line was knotted by hand with laparoscopic manipulators. The bougie was changed to a common nasogastric tube. Standard cholecystectomy was performed. The posterior wall of the duodenum was dissected from the pancreas to the line of the gastroduodenal artery. The duodenum was dissected 3–4 cm after the pylorus by cutting-closing laparoscopic tri-stapler (EndoGIA 60, Covidien, Ireland). Viability of the duodenal stump was preserved. The omentum majus was dissected vertically. The ileum (measured 300 cm back from the ileocaecal junction orally) was positioned tension-free antecolic and tied to the posterior wall of the duodenal stump by 2/0 polyglactin sutures (Surgicryl, SMI AG, Belgium). The proximal duodenal staple line was excised and a lengthwise ileotomy was made. A running hand-sewn end-to-side duodeno-ileostomy was prepared by a 2/0 polydioxanone barbed suture (V-Loc, Covidien, Ireland) and the line was secured by Ligamaxx clips (Ethicon Inc., USA) if necessary. Air–water proofing was performed. The oral part of the sewn ileum was connected to the stomach by 2/0 polyglactin sutures (Surgicryl, SMI AG, Belgium) to protect the anastomosis and reduce alkaline reflux. Intra-abdominal drainage was placed through the right upper abdominal port. The cholecyst was removed from the abdomen. During trocar removal, abdominal wall sanguinations were visualised. After exsufflation, the skin wounds were closed. A short video report is available at online depository.

### Perioperative period

Patients routinely spent the first 36–48 h after operation in ICU. On the first postoperative day, a swallowing X-ray was performed with gastrografin (Bayer Pharma AG, Germany). If there was no leak, the nasogastric tube would be removed. Patients were advised to drink by gulps, up to 200 ml on the first postoperative day, 500 ml on the second postoperative day and so on, finally reaching 2000 ml. Routinely, 6000NE enoxaparin (Clexane, Sanofi, France) injections were administered during the first month. Compression I elastic graduated stockings were applied perioperatively. Analgesics and antiemetics were part of routine treatment. Patients were advised to take oral vitamins (some kind of complex product containing types of vitamin B and D), iron supplementation (combination of 100 mg ferric (III) hydroxide polymaltose and 0.35 mg folic acid two times daily) and oral PPI (pantoprazole 40 mg one time daily) during the first postoperative month. In case of malabsorption, vitamin and iron supplementation was prolonged until serum levels were normalised or clinical signs were diminished. PPI intake was also continued in case of persistent symptoms of GERD. The postoperative diet was adapted to the change in upper gastrointestinal tract anatomy. Nutrition was set up depending on consistency of the meal during the first 8 weeks. Appropriate fluid (1500–2000 ml distributed in 200–300 ml portions per hour) and protein intake were emphasised. Initially, daily calorie intake was advised to be kept at 900–1000 kcal (protein: 73 g, carbohydrate: 73 g, fat: 43 g) which was raised to 1200 kcal (protein: 87 g, carbohydrate: 87 g, fat: 51.6 g) and 1500 kcal (protein: 109 g, carbohydrate: 109 g, fat: 64.5 g) for women and men, respectively, after the third week. Later, patients were advised to keep to a standardised diet containing equal quantities of carbohydrate and protein (30–30%) and 40% fat (1200 kcal for women, 1500 kcal for men). No added sugar was permitted. During the first 1–3 weeks, fat intake was calculated from the natural content of dishes. Afterward, the daily addition of 5–10 g of fat to food was advised. Five meals were taken every day. If necessary, meal replacements (Fresubin Protein Energy Drink, Fresenius Kabi, Ireland) or protein additives (Protifar, Nutricia, Netherlands) were permitted to complete the suggested carbohydrate, protein and fat intake. Appropriate replacement of vitamins and trace elements was necessary. After the first 8 weeks, patients were advised to keep to a balanced weight-loss diet containing equal quantities of carbohydrate, protein and fat to maintain a negative energy balance providing a deficit of 500–1000 kcal daily. The calorie requirement of each patient was calculated by Millfin St. Jeor BMR estimation formula corrected for age, gender, height, weight and activity level. Adequate weekly physical activity was advocated.

### Study size

The sample size was calculated statistically to reduce skewness. Considering a normal distribution with a statistical power of 80% and a type I error of 5%, the sample size was calculated to be 32 patients. Considering a 10% dropout rate, 35 cases were planned for inclusion in the study.

### Statistical analysis

Variables with normal distribution (analysed by Kolmogorov–Smirnov and Shapiro–Wilk tests) were presented by mean and SD; non-normally distributed variables were expressed by median and interquartile range. Categorical variables were presented as number and percentage. Means of continuous normally distributed records were analysed by two-sample *t*-test or ANOVA. The *p*-value was set to 0.05.

## Results

### Participants

**Fig. 1:**
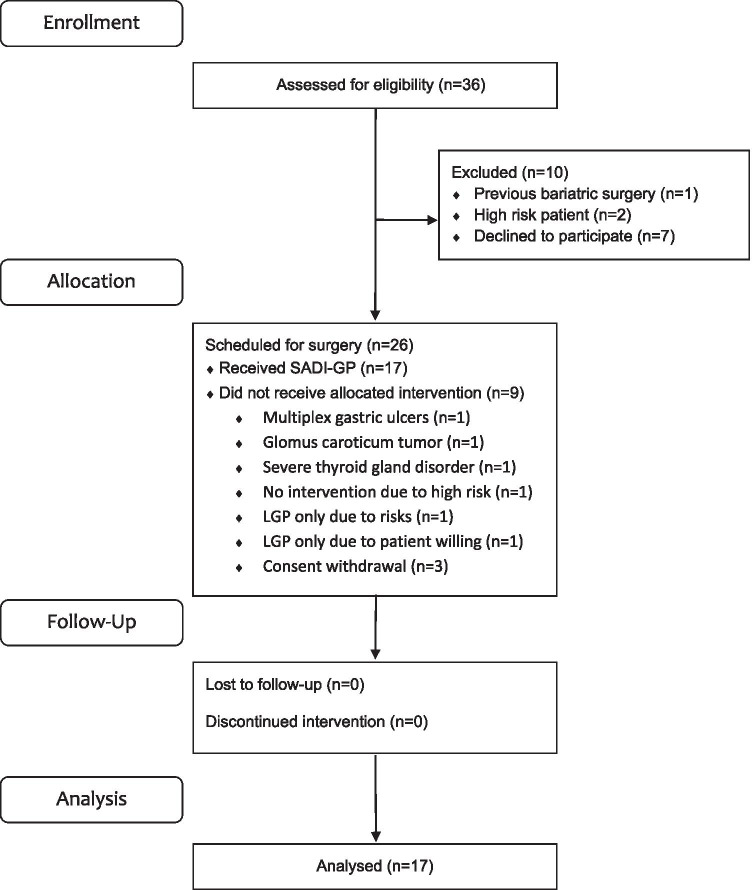
Flow chart of the study

During enrolment, 26 patients were scheduled for surgery; nine of them were excluded from the trial for different reasons, leaving 17 cases for final evaluation (15 females, mean age of included patients 40 years). Details are shown in the flow diagram (Fig. 1).

### Descriptive data

All surgeries were performed laparoscopically. On average, it took 205 min to perform SADI-GP. After 10 operations, procedures became faster. In two cases, cholecystectomy was performed earlier, but one of them had an uncomplicated Meckel diverticulum that was also resected during surgery. Only two patients had verified stones in the gallbladder before surgery, but pathological examination showed chronic inflammation in all specimens and cholesterolosis (non-neoplastic polyps) in 6 of 15 cases. Patients were observed on average for 42 h in the ICU and spent 7 days in hospital. Comorbid conditions related to obesity were frequent; the characteristics of the cases are presented in Table 1. Obesity-related comorbidities had shown favourable resolution rates (hypertension: 33%, 44.4%, 77.8% and 77.8%, dyslipidaemia: 36.4%, 36.4%, 72.7% and 81.2%, prediabetes and diabetes: 54.5%, 81.8%, 81.8% and 100% at 1-, 3-, 6- and 12-month follow-ups, respectively). Symptomatic GERD was frequent. Five patients presented gastritis at baseline, all of them were cured before surgery and there was no relapse observed during follow-up. NAFLD was present at a high rate (94.1%). In the first month, more patients presented higher levels of liver enzymes, which fell at 3- and 6-month follow-ups and remained stable. High levels of uric acid were presented by 52.9% of cases at baseline, decreasing to 41.2%, 35.3%, 25% and 7.1% at 1-, 3-, 6- and 12-month follow-ups, respectively.

**Table 1:** Characteristics of patients (*n* = 17)

*Gender ratio (female:male)*	2:15				
*Age in years presented as mean (range) [SD]*	40 (30–56) [8.13]				
*Operation time in minutes presented as mean (range) [SD]*	204.71 (150–320) [43.68]				
*Hours spent at intensive care unit presented as median (range) [interquartile range]*	42.10 (39.30–306.90) [4.55]				
*Days spent at Hospital presented as median (range) [interquartile range]*	7 (5–17) [0]				
Comorbidities	Baseline	At 1 month	At 3 months	At 6 months	At 12 months
*Hypertension*	9	6 (3 patients were without treatment)	5 (4 patients were without treatment)	2 (7 patients were without treatment)	2 (7 patients were without treatment)
*Dyslipidaemia*	11	7 (1 patient was in remission and 3 others were without treatment)	7 (1 patient was in remission and 3 others were without treatment)	3 (3 patients were in remission and 5 others were without treatment)	2 (2 patients had high serum lipid levels and 7 others were without treatment)
*Glucose metabolism*					
* Prediabetes*	2	2	0	0	0
* Diabetes*	9	6 (3 patients were in remission and 3 others were without treatment)	2 (2 patients were in remission and 7 others were without treatment)	2 (2 patients were in remission and 7 others were without treatment)	0 (all patients were without treatment)
HgbA1C % as mean* or median** (range) [SD*** or IQR****]	7.30* (6.00–8.70) [1.26]*** (*n* = 12)	6.40** (6.00–9.20) [2.45]**** (*n* = 11)	5.90* (5.30–6.50) [0.50]*** (*n* = 7)	5.48* (4.70–6.20) [0.84]*** (*n* = 4)	5.34* (5.00–5.80) [0.34]*** (*n* = 7) (*p* = 0.171)
*Upper gastrointestinal tract*					
* Gastrooesophageal reflux*	7	7	9	9	6
* Gastritis*	5	0	0	2	1
* Peptic ulcer*	1	0	0	0	0
*Non-alcoholic fatty liver disease (NAFLD)*					
* Steatosis hepatitis*	12	7	9	6 (*n* = 13)	6 (*n* = 13)
* Non-alcoholic steatohepatitis (NASH)*	3	8	5	3 (*n* = 13)	2 (*n* = 13)
* Fibrosis*	0	0	0	0 (*n* = 13)	0 (*n* = 13)
* Cirrhosis*	1	0	0	0 (*n* = 13)	0 (*n* = 13)
* Gout*	9	7	6	3 (*n* = 12)	1 (*n* = 14)
* Obstructive sleep apnoea (OSA)*	3	3	3	3	2
* Polycystic ovary syndrome (PCO)*	2	2	2	2	2
*Thyroid gland disorder*					
* Normofunction*	3	3	3	2	3
* Hyperfunction*	4	4	4	4	4
* Hypofunction*	3	3	3	4 (one patient developed subclinical hypofunction)	4
* Osteoarthritis*	5	4	3	4	4
* Cardiovascular disease*	2	2	2	2	2
* Pulmonary disease*	3	3	3	3	3
* Depression*	0	0	0	1	1
* Chronic venous disease (CVD)*	6	6	6	6	6

*Mean and SD are presented for HgbA1% at baseline and at 3, 6 and 12 months of FU. Median and IQR are presented for HgbA1C% at 1 month of FU. Statistics were computed for HgbA1C records between baseline and 12 months of FU*

### Primary outcomes

There was no conversion, death, bleeding, VTE or 30-day readmission to hospital after the surgeries. The overall perioperative complication rate was high (29.4%). There were two severe complications (11.8%). We experienced a CD4a (pulmonary insufficiency due to chronic lung disease) and a CD3b (anastomosis leakage treated laparoscopically by stitches) complication. Vomiting occurred in three cases (CD1) which represents 17.6% of patients with mild perioperative complication rate. We did not experience gastric wall prolapse or release after surgeries. All operated stomachs showed a cone shape around the cardia on the postoperative swallowing X-ray. Among late side effects, there were no clinical signs of bile reflux or narrowing of duodeno-ileal anastomosis. Mild anaemia (treated by iron supplementation) was frequent (7.1%, 33.3% and 38.5% at 3, 6 and 12 months of follow-up, respectively) in an increasing pattern of incidence but hypoproteinaemia (16.7% and 15.4% at 6 and 12 months of follow-up, respectively) was rare. Overall nutrient deficiency (17.6%, 35.7%, 66.7% and 23.1% at 1, 3, 6 and 12 months of follow-up, respectively), diarrhoea (52.9%, 41.2%, 52.9% and 23.5% at 1, 3, 6 and 12 months of follow-up, respectively) and hair loss (47.1%, 47.1% and 17.6% at 3, 6 and 12 months of follow-up, respectively) were frequent during the first 6 months after surgery, but the occurrence of these late side effects decreased remarkably by the end of the first year. Detailed results are listed in Table 2.

**Table 2:** Primary outcomes (*n* = 17)

Perioperative complications				
*Leakage*				
*Anastomosis*	1 (CD3b, treated laparoscopically by stitches)			
*Duodenal stump*	0			
*Stenosis of the duodeno-ileal anastomosis*	0			
*Bleeding*	0			
*Wound healing disorder*	0			
*Cardiovascular*	0			
*Pulmonary*	1 (CD4a, due to cor pulmonale)			
*Vomiting*	3 (CD1)			
*Gastric wall prolapse*	0			
*30-day hospital readmission*	0			
Complications classified according to Clavien-Dindo				
*1*	3			
*2*	0			
*3a*	0			
*3b*	1			
*4a*	1			
*4b*	0			
*5*	0			
Late side effects				
	*At 1 month*	*At 3 month*	*At 6 month*	*At 12 months*
*Bile reflux*	0	0	0	0
*Stenosis of duodeno-ileal anastomosis*	0	0	0	0
*Anaemia*	0	1 (*n* = 14)	4 (*n* = 12)	5 (*n* = 13)
*Overall nutrient deficiency*	3	5 (*n* = 14)	8 (*n* = 12)	3 (*n* = 13)
*Low serum level of iron*	3	3	6	2
*Low serum level of magnesium*	0	1	0	0
*Low serum level of calcium*	0	1	1	0
*Low serum level of vitamin D*	0	1	2	1
*Hypoproteinaemia*	0	0 (*n* = 14)	2 (*n* = 12)	2 (*n* = 13)
*Diarrhoea*	9	7	9	4
*Hair loss*	0	8	8	3

### Secondary outcomes

Weight-loss outcomes were very favourable representing significant improvement (two-sample *t*-test, *p* < 0.001 for all variables at 1 year) in weight loss and decrease in BMI respectively. Patients presented a mean bodyweight of 142.54 kg (mean BMI: 50.21) at baseline, which decreased to 83.96 kg (mean BMI: 29.58) at 12-month follow-up, resulting in a mean loss of 24.71%, 38.91% and 53.20% of overweight and 11.62%, 18.54%, 26.46% and 35.58% of total bodyweight at the respective follow-up timepoints. After favourable initial (at the 1- and 3-month follow-ups) results, a 37-year-old female patient presented weight regain and worsened thyroid hypofunction; however, her metabolic parameters remained within an acceptable range. Psychological assessment by using Individual Focused Cognitive Behavioral Therapy (PF-CBT) via video call revealed chronic overeating tendencies since childhood deriving from instrumental intrafamilial physical abuse and school bullying. Such unresolved traumatic events along with social isolation due to the COVID-19 pandemic and household-chore-related stress factors overloaded the subject’s coping abilities, leading to excess calorie intake. Another female patient (47 years old) regained weight (10 kg of the 34 kg lost) between the 6- and 12-month follow-ups. The reasons were disturbed microsocial circumstances, chronic disease-related care burden on the patient and foster care of the patient’s grandchild due to drug-related criminal charges against the patient’s offspring. Due to the aforementioned reasons, slow but steady loss of control over eating habits was detected and interventional PF-CBT was commenced by the second author in order to reverse the weight gain.

There was no weight regain observed among the remaining patients. Most of them experienced satisfactory weight-loss outcomes (88.2% had an EWL% over 45%). Patient’s QoL improved significantly along with the weight loss. The Weiner et al. questionnaire showed a mild amelioration (two-sample *t*-test, *p* = 0.003 at 1 year), contrary to the BAROS-Moorehead-Ardelt II questionnaire, which represented remarkable improvement (two-sample *t*-test, *p* < 0.001 at 1 year). Detailed results are listed in Table 3.

**Table 3:** Secondary outcome (*n* = 17)

Weight loss outcomes	At baseline	At 1 month	At 3 months	At 6 months	At 12 months
*Bodyweight in kg as mean (range) [SD]*	142.54 (112–195) [25.36]	125.76 (96.5–172.7) [21.1]	116.02 (89.00–155.00) [20.16]	105.84 (75.00–148.00) [20.22]	83.96 (67.00–100.00) [13.64] *p* < 0.001
*Ideal bodyweight in kg (at BMI* = *25) as mean (range) [SD]*	71 (61.6–80.1) [4.92]				
*Excess of weight in kg as mean (range) [SD]*	71.5 (35.1–124.4) [24.88]	55.3 (19.1–101.6) [20.51]	45.01 (15.10–84.40) [19.57]	34.83 (10.20–75.80) [19.07]	13.32 (2.50–27.80) [10.18] *p* < 0.001
*BMI as mean (range) [SD]*	50.21 (36.3–69) [8.73]	44.33 (31.1–61.1) [7.35]	40.84 (28.90–51.20) [6.86]	37.19 (28.90–51.20) [6.71]	29.58 (25.80–34.60) [3.43] *p* < 0.001
Excess of BMI as mean* or median** (range) [SD*** or IQR****]	25.21* (11.3–44) [8.73]***	19.29* (6.1–36.1) [7.33]***	16.43* (4.80–29.90) [6.97]***	9.60** (3.90–26.20) [11.60]****	4.58* (0.80–9.60) [3.43]*** *p* < 0.001
Weight loss presented as mean* or median** (range) [SD*** or IQR****]		15** (8–32) [6.85]****	26.51* (19.00–40.00) [6.44]***	36.70* (10.50–56.00) [10.82]***	48.86* (16.80–70.00) [20.84]***
*EWL% as mean (range) [SD]*		24.71 (11.3–45.6) [7.49]	38.91 (26.30–57.00) [7.76]	53.20 (29.90–78.40) [14.03]	75.04 (47.90–90.10) [17.09]
*TWL% as mean (range) [SD]*		11.62 (5.5–17.2) [2.88]	18.54 (14.30–23.30) [2.49]	26.46 (9.30–39.90) [7.33]	35.58 (14.90–44.30) [12.20]
*Quality of life by Weiner *et al. Questionnaire as mean* or median** (range) [SD*** or IQR****]	46.00* (38.00–53.00) [4.30]*** *n* = 17	49.07* (38.00–61.00) [5.86]*** *n* = 14	53.00** (43.50–68.00) [7.13]**** *n* = 13	56.75** (37.00–63.00) [8.25]**** *n* = 14	54.90* (40.50–66.00) [9.52]*** *n* = 9 *p* = 0.003
Quality of life by BAROS-Moorehead-Ardelt II Questionnaire as mean* or median** (range) [SD*** or IQR****]	1.41* (0.00–4.60) [1.16]*** *n* = 16	2.21* (0.00–3.80) [1.17]*** *n* = 14	3.70** (3.10–6.30) [1.70]**** *n* = 13	5.06* (0.50–8.30) [2.02]*** *n* = 14	5.96* (2.40–8.90) [2.76]*** *n* = 9 *p* < 0.001

*Mean and SD are presented for QoL by Weiner *et al*. at baseline, 1 and 12 months of FU, as well as for QoL by BAROS-Moorehead-Ardelt II at baseline, 1, 6 and 12 months of FU. Median and IQR are presented for QoL by Weiner *et al*. at 3 and 6 months of FU, as well as for QoL by BAROS-Moorehead-Ardelt II at 3 months of FU*

*Mean and SD are presented for bodyweight, ideal bodyweight, excess of weight, BMI, EWL% and TWL% at all FU timepoints and for excess of BMI at baseline, 1, 3 and 12 months of FU and for weight loss at 3, 6 and 12 months of FU. Median and IQR are presented for excess BMI at 6 months of FU and weight loss at 1 month of FU*

### MMPI-2 results

Patients were grouped based on the physiological parameter of percentage total weight loss (based on 12-month results) into the groups of those with values below 30 (TWL-30b) and above 30 (TWL-30a) and their data on the Clinical Scales, Revised Clinical Scales, Supplemental Scales, Content Scales and PSY-5 Scales compared. Independent sample *t*-tests were applied to identify any differences, which was appropriate because scores fell into the normal and high ranges on most subscales. Low scores appeared only for one subscale (High/Low scores on Cynicism); the result for this subscale is discussed separately. Based on the test scores on the physiological parameter of percentage total weight loss (based on 12-month results), five patients were included in the TWL-30b group (all participants were females; age M = 44.2; SD = 8.90) and 11 patients in the TWL-30a group (2 males/9 females; age M = 39.09; SD = 5.96). Along the Clinical Scales, a difference was found on Depression (D; *t*(13.958) =  − 2.373; *p* = 0.00; *p* < 0.05); participants in the TWL-30a group tended to show a higher score on this subscale (M = 57.90; SD = 11.57) than those in the TWL-30b group (M = 45.00; SD = 4.64). In the other subscales of the Clinical Scales there were no differences between the groups (see Suppl.1). On the Revised Clinical Scales, a significant group difference was found for Low Positive Emotions (Rc2; *t*(13.301) =  − 2.954; *p* = 0.00; *p* < 0.05); participants in the TWL-30a group showed higher scores on this subscale (M = 59.09; SD = 14.33) than those in the TWL-30b group (M = 39.40; SD = 4.56). Along the other subscales of the Revised Clinical Scales, there were no differences between the groups (see Suppl. 2). On the High/Low Scores on Cynicism (Rc3) subscale, patients showed both high and low scores, so we compared the groups using a 2 × 2 ANOVA (percentage total weight loss × High/Low Cynicism), and there was no statistically significant difference between the groups (*F*(1) = 1.156; *p* = 0.30), but there was no participant in the TWL-30b group with a low score for Cynicism (see Suppl. 3). Along the Supplemental Scales, we did not find any significant group differences (see Suppl. 4), nor along the Content Scales (see Suppl. 5). Along the PSY-5 Scales, we saw a significant difference on Introversion/Low Positive Emotionality (INTR/LPE; *t*(13.408) =  − 1.914; *p* = 0.02; *p* < 0.05), which appeared higher in the TWL-30a group (M = 57.45; SD = 15.77) than in the TWL-30b group (M = 43.40; SD = 5.18). Along the other subscales of the PSY-5 Scales, there were no differences between the groups (see Suppl. 6). Based on 12-month BMI values, patients could be categorised into subgroups of < 35 (BMI-35b), 35–40 (BMI-35–40) and > 40 (BMI-40a), and we compared their data on the Clinical Scales, Revised Clinical Scales, Supplemental Scales, Content Scales and PSY-5 Scales with One-Way ANOVA (for the three groups). Based on the BMI (based on 12-month results), 10 patients were included in the BMI-35b group (1 male/9 females; age M = 39.30; SD = 5.72); two in the BMI-35–40 (1 male/1 female; age M = 38.50; SD = 7.78) and four in the BMI-40a group (all were females; age M = 45.25; SD = 9.91). However, it should not be overlooked that the three groups were of different sizes, so the results can only be interpreted broadly. Along the Clinical Scales, there was no difference between the groups (see Suppl.7). On the Revised Clinical Scales, significant group difference was found on Low Positive Emotions (Rc2; *F*(2) = 4.037; *p* = 0.04; *p* < 0.05); participants in the BMI-35–40 group show the highest scores on this subscale (M = 68.00; SD = 8.48), followed by the BMI-35b group (M = 55.60; SD = 14.89), and the BMI-40a group showed the lowest scores (M = 38.75; SD = 4.99). We also found a significant differences between the groups on Aberrant Experiences (Rc8; *F*(2) = 4.674; *p* = 0.03; *p* < 0.05), participants in the BMI-40a group show the highest scores (M = 58.75; SD = 16.07), followed by the BMI-35–40 group (M = 50.50; SD = 2.12), and the BMI-35b group showed the lowest scores (M = 43.30; SD = 4.57). Along the other subscales of the Revised Clinical Scales, there were no differences between the groups (see Suppl. 8). On the High/Low Scores on the Cynicism (Rc3) subscale, patients showed both high and low scores, so we compared the groups using a 2 × 2 ANOVA (BMI × High/Low Cynicism), and there was no statistically significant difference between the groups (*F*(2) = 0.393; *p* = 0.67), but only some participants in the BMI-35b group had low scores on this subscale, and none in the other two groups (see Suppl. 9). Along the Supplemental Scales, we did not find any significant group differences (see Suppl. 10), nor along the Content Scales (see Suppl. 11). Along the PSY-5 Scales, we found no significant group differences (see Suppl. 12). We were able to group patients based on weight loss into the groups that lost at least 50 kg in a year (WL-50a) and who lost less than 50 kg (WL-50b) and compared their data on the Clinical Scales, Revised Clinical Scales, Supplemental Scales, Content Scales and PSY-5 Scales. Independent samples *t*-tests were applied to identify any differences, which was appropriate because scores fell into the normal and high ranges for most subscales. Low scores appeared only for one subscale (High/Low scores on Cynicism); the result along this subscale is discussed separately. Based on the test scores on weight loss, eight patients were included in the WL-50b group (1 male/7 females; age M = 42.50; SD = 8.65) and eight in the WL-50a group (1 male/7 females; age M = 38.87; SD = 5.14). Along the Clinical Scales, we did not find any significant group differences (see Suppl. 13), nor on the Revised Clinical Scales (see Suppl. 14). On the High/Low Scores on Cynicism (Rc3) subscale, patients showed both high and low scores, so we compared the groups using a 2 × 2 ANOVA (weight loss × High/Low Cynicism), and there was no statistically significant difference between the groups (*F*(1) = 0.136; *p* = 0.72), but there was no participant in the WL-50b group with a low score on Cynicism (see Suppl. 15). Along the Supplemental Scales, we did not find any significant group differences (see Suppl. 16), nor along the Content Scales (see Suppl. 17). Along the PSY-5 Scales, we did not see a significant difference (see Suppl. 18). We examined the psychological characteristics of those who lost at least 50 kg (WL-50a) in a year by Pearson correlation (IBM SPSS). This physiological parameter showed positive correlations *with* Ideas of Persecution (Rc6; *r* = 0.860; *p* = 0.00; *p* < 0.05); with Bizarre Mentation (BIZ; *r* = 0.810; *p* = 0.01; *p* < 0.05); with Self-Deprecation (DEP3; *r* = 0.714; *p* = 0.04; *p* < 0.05); with Schizotypal Characteristics (BIZ2; *r* = 0.848; *p* = 0.00; *p* < 0.05); with Explosive Behaviour (ANG1; *r* = 0.708; *p* = 0.04; *p* < 0.05); with Brooding (D5; *r* = 0.727; *p* = 0.04; *p* < 0.05); with Authority Problems (Pd2; *r* = 0.841; *p* = 0.00; *p* < 0.05); with Social Alienation (Pd4; *r* = 0.845; *p* = 0.00; *p* < 0.05); with Persecutory Ideas (Pa1; *r* = 0.826; *p* = 0.01; *p* < 0.05); with Social Alienation (Sc1; *r* = 0.748; *p* = 0.03; *p* < 0.05); and with Ego Inflation (Ma4; *r* = 0.785; *p* = 0.02; *p* < 0.05). The weight-loss parameter also showed negative correlations with Femininity/Masculinity (Mf; *r* =  − 0.716; *p* = 0.04; *p* < 0.05); with Social Responsibility (Re; *r* =  − 0.798; *p* = 0.01; *p* < 0.05); and with Gender Role-Feminine Scale (GF; *r* =  − 0.817; *p* = 0.00; *p* < 0.05; see Suppl. 19).

### Risk of bias

Sample size is smaller than expected which could lead to insecure parameters affecting complication rates and late side effects primarily. Weight loss outcomes could be biased by low number of allocated interventions at some timepoints, but 1-year records seem to be representative. MMPI-2 testing was performed only at 1-year follow-up, therefore, the results are only referential. There was no comparator to SADI-GP, thus, all of our observations are only descriptive.

## Discussion

### Key results

Our study showed that SADI-GP is a promising method but there are some concerns. We experienced only one complication related to surgery, which was treated laparoscopically. Late side effects of duodeno-jejunal exclusion were over an acceptable range. Our patients presented favourable weight-loss outcomes; in parallel, comorbid conditions related to obesity and QoL improved as expected. MMPI-2 tests showed that subjects with a weight loss of > 30 kg tended to show higher scores on the Depression (D) and Low Positive Emotions (Rc2) scales, which was supported by higher scores on Introversion/Low Positive Emotionality (INTR/LPE) scale. When compared by BMI measured at 12-month follow-up, participants in the BMI 35–40 interval group showed the highest scores on the Low Positive Emotions (Rc2) scale compared to those with BMI ≤ 35 or BMI > 40. A significant difference was found regarding the Aberrant Experiences (Rc8) scale, where participants in the BMI > 40 group showed the highest scores. A minimum weight loss of 50 kg over a year had positive correlations with the Ideas of Persecution (Rc6), Bizarre Mentation (BIZ), Self-Deprecation (DEP3), Schizotypal Characteristics (BIZ2), Explosive Behaviour (ANG1), Brooding (D5), Authority Problems (Pd2), Social Alienation (Pd4), Persecutory Ideas (Pa1), Social Alienation (Sc1) and Ego Inflation (Ma4) scales and negative correlations with Femininity/Masculinity (Mf), Social Responsibility (Re) and Gender Role-Feminine (GF) scales.

### Limitations

In general, our study has limitations due to the small sample size and study design. We did not reach the projected number of included cases because we experienced a higher-than-expected dropout rate, and the number of patients with obesity applied for screening was too low considering the strict selection criteria. There is a huge selection bias in this cohort because 88% of the study population is female. It could skew our results that there was no control endoscopic or radiologic examination scheduled for gastric plicated patients. Routine cholecystectomy was performed in 15 cases which could bias operating time and occurrence of some complications. This paper presents only short-term results; therefore, the efficacy of the procedure cannot be determined this time. Psychological evaluation lacked longitudinal data sources for comparison.

### Interpretation

LGP is not a widely applied restrictive procedure due to the necessity for expert skills in laparoscopic hand sutures and controversial results in the past [9–24]. In case series, favourable complication rates and weight-loss outcomes were observed [9–14, 17]. On the other hand, non-randomised comparative trials (most of them reporting short-term results) suggest that LGP is inferior to LSG in terms of efficacy [15, 16, 19, 21, 22] except for the RCT of Talebpour et al., because they found an initial significant difference in weight loss between the groups in favour of LSG, but this was equalised in the long term [20]. Later, Heidari et al. published a paper reporting a high rate of revisional surgery after LGP due to ineffective weight-loss management [23]. Neagoe et al. performed a comparative trial of LSG and LGP, which confirmed similar levels of safety of the methods, but LSG was superior to LGP in terms of efficacy after a 6-month follow-up. Most of the authors concluded that LGP is comparable to LSG in terms of postoperative complications; however, Albanese et al. experienced a high revision rate (60%) of LGP due to gastric wall prolapse [18]. To our knowledge, there are two meta-analyses and one systematic review available; each of them confirmed the superiority of LSG over LGP in terms of postoperative complications and weight-loss outcomes [51–53]. However, El Soueidy et al. regarded LGP as an acceptable low-budget treatment modality for obesity [54].

For a decade now, laparoscopic SADI or SADJ combined with LSG (regarded as simplified DS) has earned increasing popularity among bariatric surgeons due to its favourable complication rates and weight-loss outcomes. Sanchez-Pernaute et al. reported promising results on excellent weight-loss outcomes and favourable complication rates [28]. The key question is this: how long a biliopancreatic limb should be planned? Some authors measure the whole length of the small intestine, and afferent limb longitude is determined depending on BMI and comorbidities [39, 55]. OAGB is performed by a short 150–200-cm biliopancreatic limb [36, 37, 41, 55], similarly to SADJ [27]. Others suggest taking the common limb at least 300-cm distant from the ileocecal valve to avoid hypoproteinaemia and anaemia [28, 30, 34]. High-volume studies reporting on consecutive patients regarded SADI-SG as safe and efficient [28, 30–32, 34, 35]. Schoar et al. concluded in their meta-analysis that SADI-SG is a promising bariatric procedure regarding weight-loss outcomes, but it should be standardised due to current technical variability [29]. A meta-analysis by Lee et al., including only cohort studies, concluded that SADI-SG among BPD-DS and LRYGB is efficient as a revisional procedure after LSG [33]. There are major concerns of loop bypass, especially for OAGB, bile reflux through gastro-jejunal or -ileal anastomosis resulting in marginal ulcers and, in the worst case, oesophageal reflux leading to Barret’s metaplasia [55–57]; this is in contrast to SADI-SG and SADJ-SG, where it is reported rarely [27, 28, 30–32, 34] and results from preservation of the pylorus (functional barrier), which prevents bile reflux in physiological conditions [26].

Elbanna et al. [45] and Mohammed and Aldaaod [46] introduced laparoscopic gastro-ileal bypass with gastric plication (Mohammed and Aldaaod added pyloric plication to the method) which is similar to but not identical with our SADI-GP, because the physiological barrier of the pylorus is ceased; therefore, bile reflux to the stomach, resulting in high rates of anastomotic ulcers, should be expected. However, each short-term study reported the absence of serious biliary symptoms. There were no anastomotic ulcers observed by Elbanna et al. (sample size of 56), but it should be mentioned that Mohammed and Aldaaod experienced anastomotic ulcers in three cases (study population of 270 patients), which were treated conservatively. The studies reported low numbers of complications (two early abdominal collections treated by ultrasound-guided drainage observed by Elbanna et al. and two late perforations treated conservatively by Mohammed and Aldaaod). Side effects such as diarrhoea, anaemia and nutrient deficiencies were infrequent [45, 46]*.* Hair loss was described in 53% of patients treated by Mohammed and Aldaaod [46]*.* The trials reported favourable weight loss (EWL% ranged between 72.5 and 90%) and metabolic outcomes [45, 46]*.*

MMPI-2 tests showed that greater weight loss carries a greater probability for depression along with a lesser likelihood of positive emotional experiences; therefore, psychological support during follow-up is necessary to maintain weight loss [58]. As an introverted personality setting is examined with greater weight loss, active psychological intervention is required in order to include and maintain subjects in therapy after the surgical procedure [59, 60]. As subjects with a BMI of 35–40 score significantly higher on the Low Positive Emotions (Rc2) scale, we experienced that the transition from a BMI of > 40 towards a BMI of < 35 carries the risk of such a transition evoking anxiety and fear of the changing body. This idea is supported by the significantly high scores achieved by the aforementioned group on the Aberrant Experiences (Rc8) scale, which indicates the need for active guidance from medical professionals during this transition [61, 62].

### Comparison with other procedures

LRYGB had hegemony in bariatric surgery over the last decades. It was challenged by LSG (part of DS) because of simplicity and favourable efficacy. Perioperative mortality rates are proven to be below 0.2%. Overall, serious complications occur in less than 6% of patients for LSG and 9% of operated cases for LRYGB, respectively. Short-term reoperation rates should occur below 3% for LSG and 5% for LRYGB. The long-term TWL% of each method is around 20% [63]. The latest systematic reviews including meta-analysis comparing LYRGB with LSG proved controversial results in terms of efficacy and safety [64–66]. OAGB was described to be effective and safe compared to LYRGB [67, 68]. A meta-analysis comparing pylorus-preserving bariatric surgery to duodeno-jejunal bypass liner (endoscopic treatment modality) regarded surgical procedures (mainly SADI-SG) as safer than comparator. Mortality, reoperation rates, overall complications and severe complications for SADI-SG and SADJ-SG were 0.3%, 2.5%, 12.4% and 5.7%, respectively. Overall TWL% at 1 year was described as 36,9% (34,6–39%) which are comparable or even superior to LRYGB and LSG [69]. Our pilot study on SADI-GP has shown that mortality was zero, and there was no conversion, bleeding, VTE or 30-day readmission to hospital after the surgeries. Overall perioperative complication rate was high (29.4%). There was only one severe complication related to surgery (5.9%) similar to more widespread methods (LSG, LRYGB, SADJ-SG and SADI-SG). The 17.6% of patients needed additional treatment due to mild perioperative complications (vomiting) which was a little over of described for SADJ-SG and SADI-SG. It is suggested to keep the common limb at least 300-cm distant from the ileocecal valve to avoid hypoproteinaemia and anaemia [28, 30, 34]. We followed these instructions; however, late side effects such as mild anaemia, nutrient deficiency, diarrhoea and hair loss as a result of malabsorption occurred frequently during the first 6 months after surgery contrary to expected but the occurrence of these adverse effects decreased remarkably by the end of first year. Weight loss outcomes were at least similar to SADI-SG and SADJ-SG and superior to LRYGB and LSG. However, it should be emphasised that the overall results of our small descriptive observational study are not really comparable to complication rates and efficiency of known and widely studied methods due to high risk of bias.

## Conclusion

According to our safety study, SADI-GP is a promising pylorus-preserving malabsorptive procedure, but a long-term high-volume case series or a randomised controlled trial is necessary to evaluate complication rates and weight-loss outcomes. Surgical procedures initiate the process of weight loss, but psychological interventions must take over in order to maintain it, due to the widespread changes in the psyche and behaviour of subjects following surgical intervention. The lack of these will decrease the efficacy of weight control in the long term.

## Supplementary Information

Below is the link to the electronic supplementary material.

Supplementary file1 (XLSX 42 KB)

## Abbreviations

ANOVA: Analysis of variances
BMI: Body mass index
BMR: Basal metabolic rate
BPD: Biliopancreatic diversion
CD: Clavien-Dindo classification
CI: Confidence interval
DS: Duodenal switch
EWL%: Percentage excess weight loss
GERD: Gastroesophageal reflux disease
HgbA1C%: Haemoglobin A1C in percent
ICU: Intensive Care Unit
LAGB: Laparoscopic adjustable gastric banding
LGP: Laparoscopic gastric plication
LRYGB: Laparoscopic Roux-en-Y gastric bypass
LSG: Laparoscopic sleeve gastrectomy
NAFLD: Non-alcoholic fatty liver disease
OAGB: One-anastomosis gastric bypass
OSA: Obstructive sleep apnoea
PCO: Polycystic ovarian syndrome
PF-CBT: Problem-focused cognitive behavioural therapy
PPI: Proton pump inhibitor
QoL: Quality of life
RCT: Randomised controlled trial
SADI-SG: Single-anastomosis duodeno-ileal bypass with sleeve gastrectomy
SADJ-SG: Single-anastomosis duodeno-jejunal bypass with sleeve gastrectomy
SD: Standard deviation
T2DM: Type 2 diabetes mellitus
TWL%: Percentage total weight loss
VTE: Venous thromboembolism
